# Seven mysteries of LAG-3: a multi-faceted immune receptor of increasing complexity

**DOI:** 10.1093/immadv/ltab025

**Published:** 2021-12-20

**Authors:** Stephanie E A Burnell, Lorenzo Capitani, Bruce J MacLachlan, Georgina H Mason, Awen M Gallimore, Andrew Godkin

**Affiliations:** 1 Division of Infection and Immunity, Henry Wellcome Building, Cardiff University, Cardiff, UK; 2 Department of Gastroenterology and Hepatology, University Hospital of Wales, Heath Park, Cardiff, UK

**Keywords:** LAG-3, sLAG-3, checkpoint targets, cancer immunotherapy, structural biology

## Abstract

Despite three decades of research to its name and increasing interest in immunotherapies that target it, LAG-3 remains an elusive co-inhibitory receptor in comparison to the well-established PD-1 and CTLA-4. As such, LAG-3 targeting therapies have yet to achieve the clinical success of therapies targeting other checkpoints. This could, in part, be attributed to the many unanswered questions that remain regarding LAG-3 biology. Of these, we address: (i) the function of the many LAG-3-ligand interactions, (ii) the hurdles that remain to acquire a high-resolution structure of LAG-3, (iii) the under-studied LAG-3 signal transduction mechanism, (iv) the elusive soluble form of LAG-3, (v) the implications of the lack of (significant) phenotype of LAG-3 knockout mice, (vi) the reports of LAG-3 expression on the epithelium, and (vii) the conflicting reports of LAG-3 expression (and potential contributions to pathology) in the brain. These mysteries which surround LAG-3 highlight how the ever-evolving study of its biology continues to reveal ever-increasing complexity in its role as an immune receptor. Importantly, answering the questions which shroud LAG-3 in mystery will allow the maximum therapeutic benefit of LAG-3 targeting immunotherapies in cancer, autoimmunity and beyond.

**Technical Box**

*Surface plasmon resonance (SPR)*: A label-free technique used to determine receptor/ligand binding interactions and their strength, affinity (dissociation constant; K_D_), using purified protein samples. In SPR, one protein is immobilised to the chip surface (ligand) and potential binding partners (analytes) are flowed over the chip surface using a microfluidics system. Binding is detected in real-time due to a change in refractive index at the chip surface. Typical SPR experiments determine affinity through (i) steady-state analysis, where binding at steady state is measured across a dilution series of analyte, and/or (ii) kinetic analysis, whereby kinetic models are fitted to binding curves to determine on- and off-rates.

*BioLayer interferometry (BLI):* A similar label-free technique used to describe receptor-ligand binding interactions and determine affinities. In BLI, ligands are immobilised to a sensor probe surface which is immersed in analyte solution within a micro-well plate. Upon analyte binding, a change in the optical thickness of the probe surface is detected by a change in interference of optical white light which is reflected at the probe surface. BLI is less sensitive compared to SPR; however, higher-throughput is thus useful in searching for novel receptor/ligand interactions.

*Affinity versus avidity:* The term affinity (K_D_) describes the strength of binding between a receptor and ligand with a one-to-one binding mode. A lower measured K_D_ value (units Molar) represents a higher affinity interaction. Many one-to-one protein/protein interactions bind in the micromolar (µM) range. Molecules such as antibodies, and LAG-3:Fc, are multimeric (IgG and LAG-3:Fc = dimeric) and, as a result, are able to engage multiple ligands. Consequently, multimeric molecules utilise avidity effects to more strongly engage ligands (typically with avidity values in the nanomolar to the picomolar range). Dimeric molecules can engage a second ligand species, thus prolonging off-rates. Avidity effects are dependent on the availability of multiple ligands on an attached surface (such as the cell surface or experimental chip or plate). It is important to remember that LAG-3:Fc has been *artificially* dimerised through the covalently linked Fc domain and thus the avidity effects are not representative of LAG-3 in a cellular context. Careful evaluation of experimental setup is required when evaluating the strength of LAG-3-ligand interactions using LAG-3:Fc.

## Introduction

Lymphocyte activation gene 3 (LAG-3) is a single-pass transmembrane glycoprotein, which despite 30 years of research, continues to be elusive. The majority of LAG-3 research has focused on its function on conventional T cells, where it is generally understood to negatively regulate the immune response, for example, by inhibiting proliferation and reducing granzyme/cytokine production [1–3]. Its role in modulating the inhibitory function of regulatory T cells (Tregs) on the other hand is contentious, with conflicting evidence suggesting LAG-3 either limits or contributes to Treg inhibition [4–6]. Perhaps the next most recognised role of LAG-3 is its involvement in TLR-independent activation of plasmacytoid dendritic cells (pDC) [7]. LAG-3 expression has also been described on other immune cells, including B cells, NK (natural killer) cells and unconventional T cells; upon which the function of LAG-3 is less well understood [1,8–11].

Despite these significant knowledge gaps, LAG-3 is often referred to as the ‘next immune checkpoint’ target on lymphocytes [12–14]. The proposed targeting of LAG-3 in immunotherapies has taken many forms, including (i) the delivery of soluble dimeric LAG-3 as an adjuvant therapy [15], (ii) the antibody blockade of LAG-3 interactions with its ligand(s) in cancer which has also been combined with an anti-Programmed Cell Death Protein 1 (PD-1) targeting therapy [16], (iii) antibody-mediated depletion of LAG-3^+^ cells in autoimmunity [17] and (iv) modulation of LAG-3 expression through small molecule targeting of Glycogen synthase kinase-3 (GSK-3) in cancer [18]. Despite ongoing efforts, LAG-3 targeting therapies are yet to achieve the successes of PD-1 and cytotoxic T-lymphocyte associated protein 4 (CTLA-4) [4], despite being identified around the same time [19–21]. The targeting of LAG-3 in combination with other checkpoint therapies has been a focus of ongoing clinical trials – exemplified by a recent study that associated LAG-3 expression on peripheral CD8^+^ T cells with a poorer efficacy of checkpoint blockade in melanoma and urothelial carcinoma patients [22]. Here, we address seven mysteries of LAG-3 and discuss the hurdles that remain on the path to maximizing its therapeutic potential.

## LAG-3 ligands: not just MHC-II?

**Figure 1.**
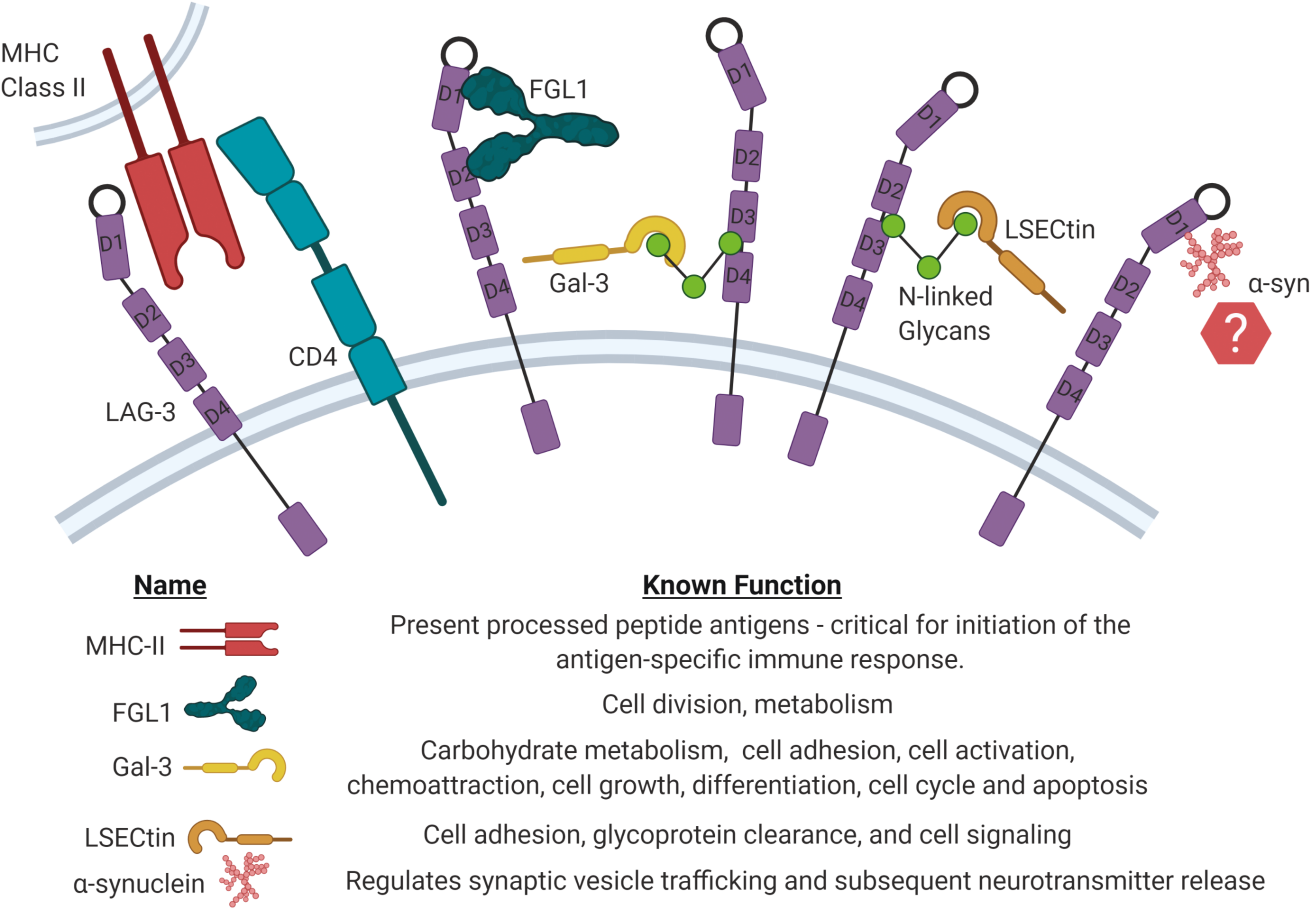
Possible LAG-3 ligands and their functions; MHC-II, FGL1, Gal-3, LSECtin, and α-synuclein. The interaction between LAG-3 and MHC-II is believed to occur between the D1 loop of LAG-3 binding to a membrane-proximal site on MHC-II, similar to the defined MHC-II/CD4 binding site which is shown here for reference. FGL1 binds to LAG-3 at two sites in D1 and D2, while Gal-3 and LSECtin bind to N-linked glycans at glycosylation sites and α-syn has been shown to bind to the D1 domain of LAG-3.

Upon first identification, parallels between LAG-3 gene structure and CD4 [23] led to an immediate investigation into major histocompatibility complex class II (MHC-II) as a potential ligand. LAG-3 expressing COS-7 cells engaged MHC-II bearing B lymphocytes which could be blocked by either anti-LAG-3 (clone 17B4) or anti-pan-HLA-II (clone D1.12) antibodies [24]. Today, however, multiple other binding partners have been proposed including liver and lymph node sinusoidal endothelial cell C-type lectin (LSECtin/CLEC4G), Galectin3 (Gal-3), Fibrinogen-like protein 1 (FGL1), and α-synuclein (α-syn) (Fig. 1) [25–27].

### MHC-II: the canonical ligand of LAG-3

**Figure 2.**
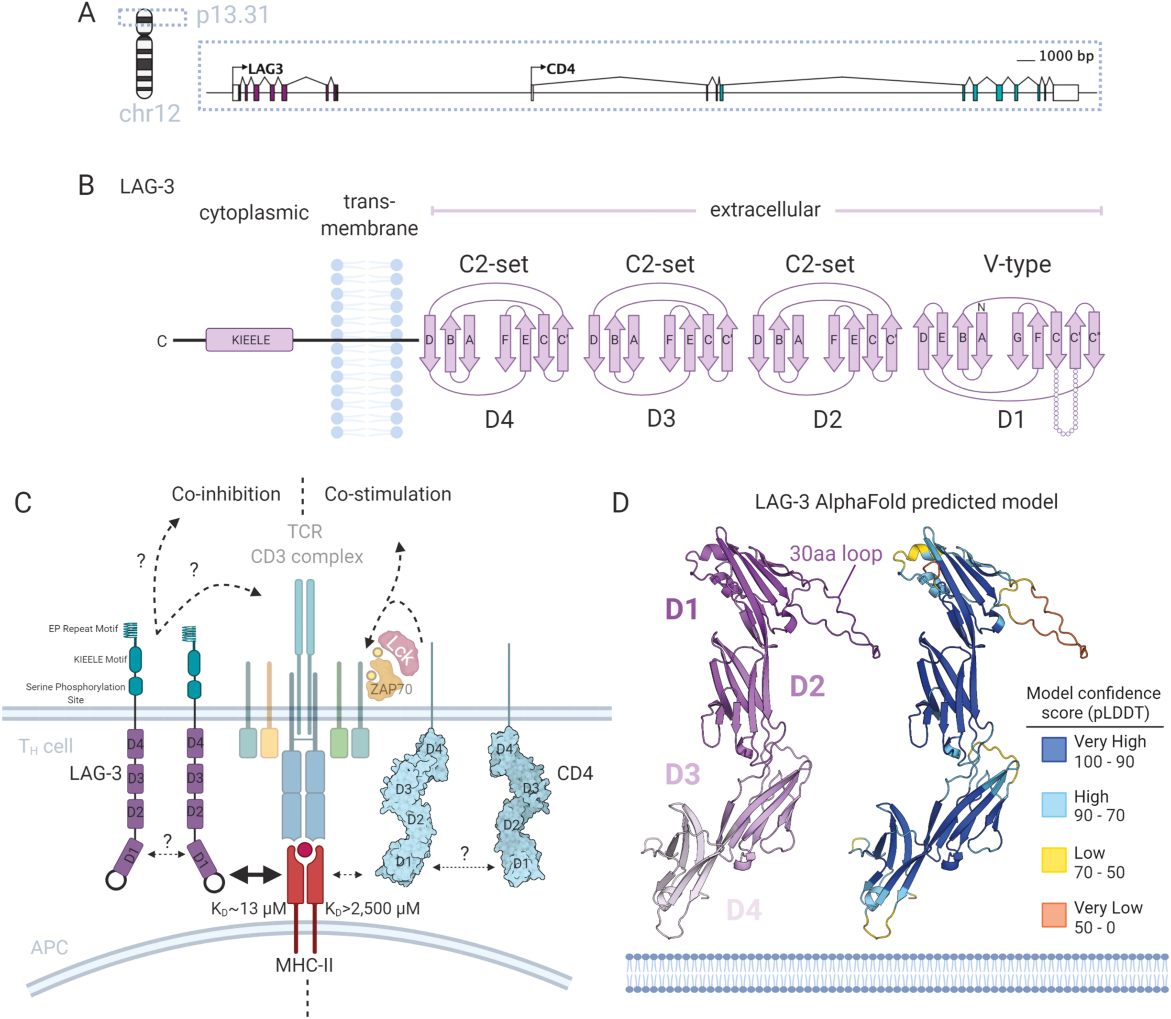
Molecular understanding of LAG-3 binding to MHC-II as a co-inhibitory competitor to CD4. (A) Gene structure and location of LAG-3 on chromosome 12 adjacent to CD4. (B) The predicted protein domain folds of LAG-3 containing four extracellular Ig-domains (D1–D4) of V-type (D1) and C2-set (D2–D4). LAG-3 has a single-pass transmembrane domain and a short cytoplasmic tail containing a unique KIEELE motif involved in signal transduction. (C) Model of LAG-3 co-inhibitory competition with CD4 whereby LAG-3 binds MHC-II at a higher affinity than the co-stimulatory CD4 and transducing inhibitory signalling through undescribed signalling pathways. (D) Model of the LAG-3 extracellular domain as predicted by the AlphaFold project. The LAG-3 model is shown as cartoon representation and coloured (left) by domain (colours indicated inset) and (right) model confidence score (pLDDT) with colours indicated by the inset legend table. pLDDT confidence levels and definitions are as described previously [31].

Like the CD4 co-receptor, LAG-3 is located on chromosome 12 (12p13.1) and also has four extracellular immunoglobulin superfamily (IgSF) like domains (D1-4) (Fig. 2A and B). Molecular characterisation of LAG-3 binding to MHC-II molecules (and the discovery of novel ligands) has been hindered by difficulties in producing soluble LAG-3 protein. The formulation of LAG-3 as a soluble fusion protein, whereby D1-4 were fused to the fragment crystallisable (Fc) domain of IgG1 [28, 29], known as LAG-3:Fc, or as a pentabody [30], remains the only documented formulation for soluble LAG-3 protein production and have been used to confirm binding to MHC-II [29]; an interaction with greater avidity than equivalent MHC-II/CD4:Fc fusion dimers [28].

The LAG-3/MHC-II interface was subsequently attributed to a proline-rich 30 amino acid (a.a.) loop, not present in CD4, between the C and Cʹ β strands of the LAG-3 D1 domain, as the anti-LAG-3 (clone 17B4) antibody raised against the 30 a.a. loop blocked the interaction [24]. Later, LAG-3:Fc mutants were designed and tested for their ability to bind MHC-II molecules in a cell adhesion assay which again identified a cluster of residues at the base of the 30 a.a. loop which disrupted binding [32]. Interestingly, implication of the 30 a.a. loop in binding suggests a smaller binding surface area than the interaction between CD4 and MHC-II [33].

More recently, using LAG-3-EC (a pentabody of mLAG-3), enhanced binding to the mouse MHC-II molecules I-A^d^, I-A^b^, and I-A^g7^ was observed when loaded with stable ­peptides compared to mutant peptides known to decrease MHC-II-peptide stability [30], which influenced the strength of ­inhibition through LAG-3 in the OT-II T cell receptor (TCR) system. Consequently, it was hypothesised that LAG-3 is selectively tuned to recognise MHC-II bearing high-affinity epitopes and mediate peripheral tolerance to high-affinity binding self-peptides. Although preferential binding of MHC-II allotypes has not been established, LAG-3:Fc was shown to bind a panel of HLA-DR, -DQ, and -DP molecules in a bead-based binding assay [34]. Thus, the known MHC-II ligands of LAG-3 were extended to 95 HLA-II molecules – all allotypes tested. Differences in affinity between LAG-3 and the HLA-II panel, however, were not examined.

LAG-3 has often been described as a high-affinity ligand for HLA-II owing to an apparent calculated affinity constant (K_D_) of 60 nM determined by titrated LAG-3:Fc binding to HLA-II expressing cells [28]. An accurate monovalent affinity of the LAG-3/HLA-II has been difficult to obtain however due to the difficulties in producing monomeric LAG-3 (see *Technical box*). Consequently, our lab has recently used surface plasmon resonance (SPR) (see *Technical box*) to directly measure binding between HLA-DR1 and LAG-3:Fc. Using titrated amounts of immobilised proteins on the sensor chip, we estimated the monovalent interaction between HLA-DR1 bearing a high-affinity peptide (Influenza A Haemagglutinin_306–318_) and LAG-3 to be in the low micromolar range (K_D_ ≈ 13 µM) [35]. This affinity measurement is in a similar range as HLA-I binding co-inhibitory receptors, such as the ILT/LILRB family [36] but still significantly stronger affinity than that of HLA-II/CD4 which is estimated at K_D_ = 2.5 mM [37]. These findings lend further support to the idea that LAG-3 can out-compete CD4 for HLA-II binding (Fig. 2C).

### A new key ligand?

There are several anti-LAG-3 antibodies, with different MHC-II-blocking abilities. Mouse studies have largely used anti-mouse LAG-3 clone C9B7W, which binds to D2 of mouse LAG-3 (mLAG-3) and weakly attenuates the interaction between mLAG-3 and MHC-II [38]. Whereas many human studies have used clone 17B4, which binds to D1 (raised to the 30 a.a. loop), and strongly blocks binding to MHC-II [24]. Interestingly a mouse study that characterised blockade by three antibodies, which bound to either D1, D2, or D4 of LAG-3, found there was no difference in T cell activation between the D1 and D2 binding antibodies [38, 39]. This suggests that even partial blockade of the LAG-3/MHC-II interaction increases T cell activation. However, it is also possible that there exists another ligand for LAG-3, which may be strongly blocked by D2 binding antibodies, but not D1 binding antibodies.

In 2019, FGL1 was described as another ligand for LAG-3 in both human and mouse [27]. FGL1 was identified as a candidate ligand first using a semi-automatic gene expression and detection system. FGL1 shares a similar structure with fibrinogen beta and gamma but has no known role on platelets or in clot formation [40]. FGL1 is primarily secreted in low levels by hepatocytes and is thought to play a role in cell division and metabolism [41–45]. However, Wang *et al.* identified upregulation of FGL1 mRNA in some solid human tumours, despite being downregulated in others. The Cancer Genome Atlas (TCGA) indicates that FGL1 is upregulated in lung adenocarcinoma [27]. It is possible that the engagement of LAG-3 by FGL1 in the tumour micro-environment prevents an effective anti-cancer immune response.

Direct interaction was shown using BioLayer interferometry (BLI) analysis (see *Technical box*) using mFGL1 and mLAG-3:Fc, with a K_D_ value of ~1.5 nM. This is an extremely high-affinity interaction (see *Technical box*), similar to an antibody that reflects multivalency of the mLAG-3:Fc to mFGL1. mLAG-3 mutagenesis studies by Wang *et al.,* found mLAG-3 bound to the fibrinogen-like domain (FD) of mFGL1. Furthermore, the single point mutation (Y73F) in the Cʹ strand of mLAG-3 D1 domain, which was previously shown to disrupt MHC-II binding, did not prevent mFGL1 binding [27]. In addition, pre-incubation of LAG-3^+^ 293T cells with C9B7W, led to complete abrogation of mouse FGL1/LAG-3 binding. These data support the hypothesis that FGL1 is an additional ligand for LAG-3 which binds to a site distinct to MHC-II. It will be interesting to investigate whether dual treatment of anti-LAG-3 antibodies which strongly block both MHC-II and FGL1 improves anti-tumour immunity compared to monotherapy, or if there are no benefits from inhibiting both ligands.

### LAG-3: more than just protein binding

LAG-3 is a glycosylated protein comprising N-linked glycans (branched glycans attached via a nitrogen atom of Asn residues at Asn-X-Ser/Thr motifs) [24, 46]. Lectins, glycan-binding proteins which include C-type lectin receptors (CLRs), siglecs, and galectins, are able to specifically interact with and select for these carbohydrate structures.

LSECtin, a member of the DC-SIGN family, is a Ca^2+^-dependent CLR shown to interact with mannose, *N*-acetyl glucosamine (GlcNAc), and fructose and is highly expressed in the liver [47]. Evidence that LSECTin limits T-cell activity was obtained using mouse models of acute viral infection [25, 48, 49]. To date, only a single study provides evidence that LSECTin can bind to N-glycans on LAG-3. Xu and colleagues, using SPR (see *Technical box*) and co-immunoprecipitation assays, showed that LSECtin was able to interact with mLAG-3, and this interaction could be blocked by treatment with C9B7W, which restored IFN-γ secretion [25]. Further studies are needed however to establish LSECTin as a physiological LAG-3 ligand in humans.

Galectins are small soluble proteins with one or two carbohydrate-binding domains specific for galactose-containing glycans [50]. Gal-3 (31 kDa) differs from other galectin family members having both a carbohydrate recognition domain and an oligomerisation domain that enables cross-linking of its binding targets. Gal-3 binds to highly branched N-glycans on extracellular matrix glycoproteins (such as CD29) and has been shown to regulate T-cell activation [51, 52]. Kouo *et al.,* used co-immunoprecipitation assays to demonstrate that Gal-3 is bound to LAG-3 [26] and hypothesised that Gal-3 binding to LAG-3 forms cross-links resulting in an inhibitory signal and thus suppression of T cell function. A recent study using multiple myeloma patient bone marrow mononuclear cells demonstrated higher rates of proliferation when treated with an anti-Gal-3 antibody compared to a pan HLA-II antibody [53]. Characterising anti-LAG-3 epitopes and their capacity to block different LAG-3 ligands would help decipher the role of different LAG-3-ligand interactions and the consequences of their blockade on T cell function.

Intriguingly, LAG-3 expression has been identified in the brain and binding to α-syn, a presynaptic neuronal protein, has been postulated (Fig. 1). α-Syn does not have a defined structure, has been shown to localise to the presynaptic terminals, and is thought to be a modulator of synaptic transmission [54]. This interaction was investigated by Mao *et al.,* using a mouse model of Parkinson’s disease (PD) [55]. Dual treatment with C97BW (D2 binding) and 410C9 (D3/D4 binding) mAb clones were shown to block α-syn pre-formed fibril (PFF) binding, endocytosis, and pathology. The potential role of LAG-3 in the brain and potential binding to α-syn is discussed in more detail in the section *Is LAG-3 expressed in the brain?*

Having multiple ligands, some of which have been shown to bind non-redundantly, may enable LAG-3 to modulate the immune response in different ways. The fact that there are both soluble and membrane-bound ligands adds another layer of complexity and control. In summary, there is still much work needed to understand the effects of LAG-3 engaging one or more of its ligands and which treatment strategy is most suited in different disease states.

## What is the molecular structure of LAG-3?

Structural understanding of protein/protein or receptor/ligand interactions can be complementary in designing approaches for interfering with these interactions, such as *in silico* screening of candidate small molecules. So far, the structure of LAG-3 is unknown, so this above avenue has not yet opened. Single-pass transmembrane proteins such as LAG-3 are usually expressed for structural studies as truncated extracellular domains to remove (i) the hydrophobic transmembrane domain which acts as a barrier to solubility and (ii) the cytoplasmic signalling domain which is often highly mobile [56, 57].

Furthermore, heavily glycosylated proteins, such as LAG-3, are problematic for structural analysis due to the randomness or variability of glycosylation patterns [58]. So far, the expression of suitable LAG-3 protein for structural studies has proven challenging. Although LAG-3:Fc fusion proteins have been expressed and proven useful for LAG-3 ligand discovery, these fusion proteins are difficult to crystallise. Even cutting-edge technologies which open cryo-electron microscopy (cryo-EM) to smaller proteins – such as Volta phase plates [59, 60], and protein scaffolds [61] have yet to materialise more detailed structural analyses of LAG-3.

Recently, a predicted model of LAG-3 was proposed by the AlphaFold project [62] whereby structure predictions for all human proteins were performed and made available [31] (Fig. 2D). Here, agreement with the IgSF domain arrangement first proposed in 1990 by Triebel *et al.* [23] (Fig. 2B) was similarly predicted. Likewise, the adjunct 30 a.a. extra loop between the C and Cʹ anti-parallel β-sheets of D1 was also observed in the prediction. This loop, however, exhibits very low positional confidence likely owing to sequence novelty and resultant lack of templates in the prediction model. As a result, a molecular understanding of how LAG-3 engages ligands remains unsolved and, at present, experimental structural data of LAG-3 is limited to a crude low-resolution envelope of LAG-3:Fc [63].

## How does LAG-3 transduce a signal?

Understanding the signal processes which regulate LAG-3 expression and its functionality as an immune receptor is essential in describing the full breadth of LAG-3 activity and unlocking the potential for therapeutic targeting. The cytoplasmic tail of LAG-3 was shown to be indispensable to LAG-3 mediated inhibition of IL-2 production [64], highlighting that signal transduction through LAG-3 drives inhibitory function. The 54 amino acid cytoplasmic domain, however, is unique as it does not encode any of the classical inhibitory phosphatase binding motifs found amongst other immune-modulatory receptors e.g. immunoreceptor tyrosine-based inhibitory motifs (ITIMs). The sequence does, however, contain three characteristic features first highlighted through conservation between human and mouse LAG-3: (i) a potential serine phosphorylation motif (S454), (ii) a repetitive segment consisting of a glutamic acid-proline dipeptide (EP motif) and (iii) a unique KIEELE motif [64].

Of these three features, the KIEELE motif was initially identified as the key driver of LAG-3 signal transduction through truncation experiments whilst S454 was seemingly less important [64]. More recently, however, using an *in vitro* T cell activation system with LAG-3-MHC-II blocking mAbs, it was shown that loss of the KIEELE sequence did not affect the inhibitory function of LAG-3 [38]. Instead, T-cell activation was altered via two other distinct mechanisms. Firstly, inhibitory function was honed to an additional region proximal to the membrane termed the FxxL motif; implicating residues F475 and L478 in directly mediating IL-2 inhibition. Secondly, whilst LAG-3^+^ cells deficient for the EP motif (also termed EX repeat) initially demonstrated similar inhibitory capacity as full-length LAG-3, mutating LAG-3 to lack both the FxxL motif and truncating the EP motif instead completely abrogated inhibition and, in fact, rendered LAG-3 co-stimulatory [38]. This suggested that the membrane-proximal region containing the FxxL motif plays the greater inhibitory role [38]. The FxxL motif is more reminiscent of the ITIM motif YxxL and thus it is hypothesised that intracellular signalling factors may recruit to this motif [38]. Whilst unknown for FxxL, an intracellular factor has been identified for the EP motif which was shown to directly recruit the LAG-3-associated protein (LAP) [65]. The downstream signalling pathways associated with these motifs and the full repertoire of signalling molecules associated with LAG-3, however, remain unsolved.

## Does soluble LAG-3 have a function?

**Figure 3.**
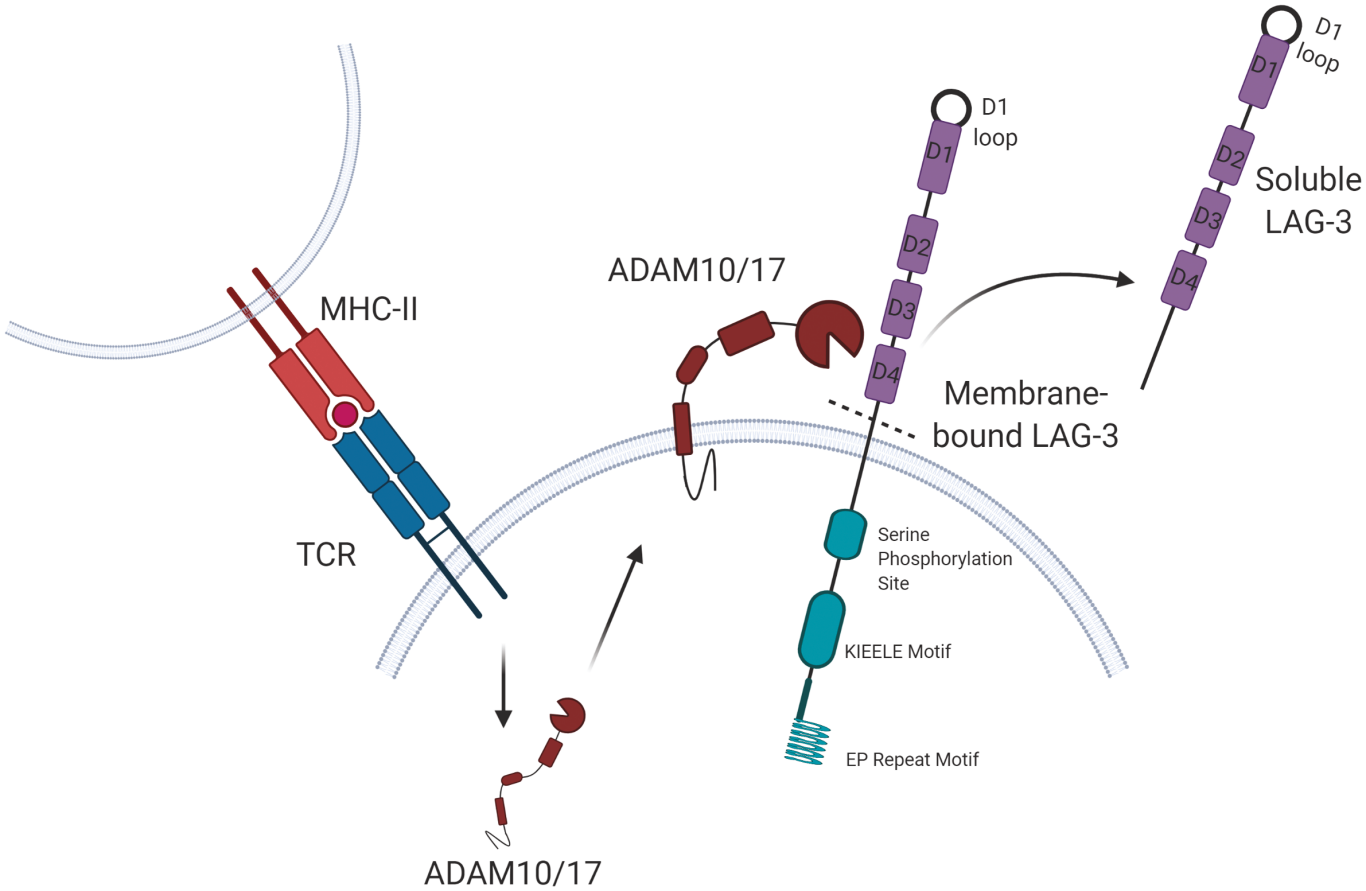
Cleavage of LAG-3 from the cell membrane. Soluble LAG-3 is produced when membrane-bound LAG-3 is cleaved by matrix metalloproteinases ADAM10 or ADAM17 between the D4 and transmembrane domains. The role of sLAG-3 is still unknown.

Cell surface LAG-3 is regulated, in part, via cleavage of the extracellular domain, resulting in a 52 kDa soluble form of LAG-3, known as soluble LAG-3 (sLAG-3). Cleavage is mediated by two TCR-induced metalloproteases, a dis-integrin and metalloproteinase domain-containing proteins 10 and 17 (ADAM10 and ADAM17) (Fig. 3) [66, 67]. Although sLAG-3 has been investigated in many chronic disease settings such as cancer and neurodegenerative diseases, no clear biological function has been identified [68–72]. It is unclear whether increased serum sLAG-3 levels reflect an increase in LAG-3 expression and turnover, or whether an increase in cleavage of cell surface LAG-3 plays a particular role in modulating its functions. Expression of sLAG-3 has been attributed, *in vivo,* to activated T cells [66] but also pDCs which produce potentially five-fold more sLAG-3 than activated T cells [9]. Given the expression of membrane-bound LAG-3 on both B cells [8] and NK cells [73], these cell types may also produce the soluble form.

### Soluble forms of other immune checkpoints

Evidence demonstrates that sLAG-3 is produced via cleavage from the cell surface or from alternatively spliced RNA [66, 67, 74]. Several co-inhibitory molecules such as PD-1, PD-L1, and CTLA-4 also have both membrane-bound and soluble forms [75]. Studies have suggested that soluble PD-1 mediates an immune-modulating effect in part by binding to membrane-bound PD-L1, obstructing the interaction of membrane PD-1 and PD-L1, counteracting the inhibitory effects of this interaction [76, 77]. On the other hand, soluble PD-L1 has also been shown to bind to PD-1 on the surface of T cells, inducing an inhibitory signal that prevents T cell activation and proliferation. Soluble CTLA-4, secreted during an immune response, has also demonstrated potent inhibitory properties [78, 79]. Whilst the contribution of these soluble forms to immunosuppression is not well understood, it is reasonable to postulate that sLAG-3 may perform a similar role.

### Does cleavage of LAG-3 serve more than a regulatory function?

Li *et al*. showed that cleavage of LAG-3 from the cell surface increases T cell function yet sLAG-3 had no effect on antigen-driven T cell activation and proliferation [67]. Further to this, the same group showed that the half-life of passively transferred, purified sLAG-3 was less than four hours, and that sLAG-3 did not specifically bind to MHC-II or B cells. This led to the hypothesis that once LAG-3 is removed from the cell surface, it is degraded or secreted serving no further function [67]. In summary, it still needs to be determined whether sLAG-3 is purely a short-lived bi-product of T cell activation or whether it serves a specific immune-modulating function whether this is inhibitory or stimulatory as discussed below.

### A role for sLAG-3 in prognosis?

Despite uncertainties regarding function, serum sLAG-3 has been investigated as a prognostic marker, particularly in cancer. In non-small cell lung cancer (NSCLC), serum sLAG-3 was inversely correlated with stage; levels were significantly higher in stages I–II NSCLC than in stages III–IV [68]. Additionally, high serum sLAG-3 level at diagnosis for breast cancer patients was associated with longer disease-free survival after a long follow-up [70]. When serum sLAG-3 levels were examined in patients with gastric cancer (GC), its expression correlated with TNM stage, depth of tumour invasion and degree of tumour differentiation in addition to positively correlating with IL-12 and IFN-γ levels [69]. This study also demonstrated that the administration of recombinant sLAG-3 in GC-bearing mice prolonged overall survival, increased survival rate and increased the CD8^+^ T cell response. The authors speculated that this was due to a greater expansion of antigen-specific CD8^+^ T cells [69]. Hence, in certain solid epithelial cancers, the presence of sLAG-3 is associated with a favourable prognosis. Despite identifying sLAG-3 levels in the sera of patients in these disease settings, identification of the cell types involved in producing sLAG-3 is lacking owing to the difficulties of tracing back to the cells responsible for this measured sLAG-3. However, in patients with chronic lymphocytic leukaemia (CLL), where sLAG-3 levels were higher in patients whose disease had progressed compared to those with stable CLL, sLAG-3 production was able to be attributed to the malignant B cells themselves [80]. Here, sLAG-3 was shown as a marker of tumour burden by tracking mRNA expression of the soluble isoform of LAG-3 (LAG-3V3) as opposed to the cleaved soluble form.

In another study, increased LAG-3 expression on CD4^+^ T cells was shown to correspond with high bacterial burden and active tuberculosis (TB) infection [81]. A follow-up study indicated that sLAG-3 levels increased during treatment, corresponding to treatment response and patients with only small increases in sLAG-3 levels during treatment demonstrated poor clinical outcome and/or failed to respond to their treatment [82]. These two studies taken together imply that during TB treatment, LAG-3 is cleaved from the cell surface at an increased rate, due to the increased activity of Th1 cells. Moreover, sLAG-3 levels in the plasma may act as a marker of treatment efficacy in TB.

### Use of sLAG-3 in therapy

LAG-3 based therapy has employed the use of the LAG-3:Fc dimer, described previously, comprising the extracellular Ig domains of LAG-3 joined to the Fc section of an antibody [29]. Eftilagimod alpha (also known as LAG-3:Fc or IMP321) stimulates dendritic cells (DCs) via MHC-II. This results in an enhanced presentation of antigen to T cells and as such is classified as a member of the new class of adjuvant/antigen-presenting cell (APC) activators, use of which may result in a better response to vaccination [83–85]. During an early phase I study in 2007, patients co-injected with Eftilagimod alpha and the Hepatitis B vaccine (HBsAg) demonstrated a lower incidence of adverse events, faster and higher antibody responses, and an increased number of vaccine responders when compared to the group injected with the vaccine alone [83]. Eftilagimod alpha has been used as an adjuvant for treatment strategies in a range of phase I studies, including renal cell carcinoma, breast cancer, melanoma, NSCLC, and metastatic HNSCC – enhancing immune response and anti-tumour activity with little to no toxicity [70,83,86]. Additionally, a phase IIb clinic trial, that ended in December 2020, investigating Eftilagimod alpha with paclitaxel in patients with metastatic breast cancer presented with favourable results (https://www.globenewswire.com/news-release/2020/03/25/2005929/0/en/Immutep-Reports-Supportive-Efficacy-Data-from-the-Phase-IIb-AIPAC-Study-Overall-Survival-Data-Expected-in-Late-2020.html) [85]. There are multiple LAG-3 therapies in the clinic for a range of diseases, Table 1 examines those that are complete.

**Table 1.** Clinical trials (complete, withdrawn, terminated, or suspended) that have involved the use of LAG-3 as a therapeutic either alone, in combination, or as an adjuvant therapy

Identifier	Therapeutic (LAG-3 mechanism)	Disease	Phase	Participants	Status	Results
NCT00354861	sLAG-3 (IMP321/eftilagimod alpha) an *APC activator (sLAG-3:Fc)* with hepatitis B antigen	Healthy volunteers (male)	1	48	Completed February 2006	No study results have been reported
NCT00354263	sLAG-3 (IMP321/eftilagimod alpha) alone or as an adjuvant to a reference flu antigen	Healthy volunteers (male)	1	60	Completed February 2006	No study results have been reported
NCT00351949	sLAG-3 (IMP321/eftilagimod alpha)	Metastatic renal cell carcinoma	1	24	Completed October 2008	No clinically significant adverse events were observed. Tumour growth reduced and PFS better in patients with higher doses [84]
NCT00349934	sLAG-3 (IMP321/eftilagimod alpha) with paclitaxel (chemotherapy)	Metastatic breast cancer	1	33	Completed January 2010	Increased number and activation of APC and percentage of NK and effector memory CD8 T cells. Clinical benefit observed in 90% of patients [87]
NCT00732082	sLAG-3 (IMP321/eftilagimod alpha) with gemcitabine (chemotherapy)	Pancreatic cancer	1	18	Terminated September 2012	Company manufacturing study drug was unable to continue production
NCT00365937	sLAG-3 (IMP321 with eight HLA-A2 restricted peptides	Melanoma	1/2	19	Terminated December 2013	New regulations of the peptides by the pharmaceutical company
NCT01308294	sLAG-3 (IMP321/eftilagimod alpha)	Melanoma	1/2a	16	Terminated April 2014	Low enrolment rate. Side effects were mild to moderate. Specific CD4 T-cell responses found in all 16 patients. Conclude that vaccination with IMP321 is promising and safe and induces sustained immune responses [88]
NCT02195349	Anti-LAG-3 (GSK2831781) *IgG1 antibody-dependent cell cytotoxicity (ADCC) enhanced mAb – depleting*	Healthy volunteers and patients with plaque psoriasis	1	67	Completed March 2018	No safety or tolerability concern was identified following a single IV dose of GSK2831781 up to 5 mg/kg. Psoriasis disease activity improved compared to placebo [17]
NCT02676869	sLAG-3 (IMP321/eftilagimod alpha) and pembrolizumab (Anti-PD-1)	Metastatic melanoma	1	24	Completed December 2019	No study results have been reported
NCT03965533	Anti-LAG-3 (GSK2831781) *Depleting antibody*	Healthy volunteers	1	37	Completed December 2019	No study results have been reported
NCT03489369	Anti-LAG-3 (Sym022) *Fc-inert mAb -antagonistic*	Metastatic cancer, solid tumour or l ymphomas	1	15	Completed January 2020	No study results have been reported

With the information we have to date, it is clear that further work is urgently required to determine the physiological role of sLAG-3 and its utility as an immunotherapeutic agent. Importantly, the lack of progression of LAG-3 monotherapies, despite a relative lack of toxicity issues, may indicate a potential redundancy between co-inhibitory receptors (as explored further below). This also highlights the need to explore combination blockade further as a possible key to unlocking the potential of LAG-3 targeting therapies.

## LAG-3 knockouts: where is the phenotype?

Perhaps indicative of the modest effect of LAG-3 blockade as a monotherapy in human cancers [89] and mouse tumour models [90, 91] complete knockout of LAG-3 in mice displayed normal immune function [10] – in stark contrast to the lymphoproliferative disease observed in CTLA-4 knockout mice [92]. First described by Miyazaki *et al.* [10], homozygous LAG-3 mutant mice appeared healthy, displayed normal T cell thymic development, and possessed typical numbers of peripheral CD4^+^ and CD8^+^ T cells with expected behaviours [10]. Subsequent studies showed that after 5 weeks of age, LAG-3^−/−^ mice possessed approximately twice the number of αβ T cells with no impact on T cell phenotype or the ratio of T cell types (naïve, memory, or regulatory) [93]. LAG-3^−/−^ mice also had higher numbers of other immune cells, including γδ T cells, NK cells, B cells, macrophages, and DCs [93]. Given that one of the key characteristics of LAG-3 is its ability to inhibit T cell proliferation, the increased number of T cells in LAG-3^−/−^ mice is unsurprising [1, 4]. Accordingly, during adoptive transfer experiments, the genetic ablation of LAG-3 results in enhanced homeostatic expansion of LAG-3^−/−^ T cells compared to wild-type (WT) T cells [93, 94]. This is in contrast to recent observations that CAR-T cells lacking LAG-3 were not functionally distinct to their LAG-3 expressing counterparts [95]. Despite this, LAG-3 along with PD-1 and TIM-3 was reported to define a subpopulation of T cells positively associated with clinical response in CAR-T therapy in patients with large B cell lymphoma [96].

**Figure 4.**
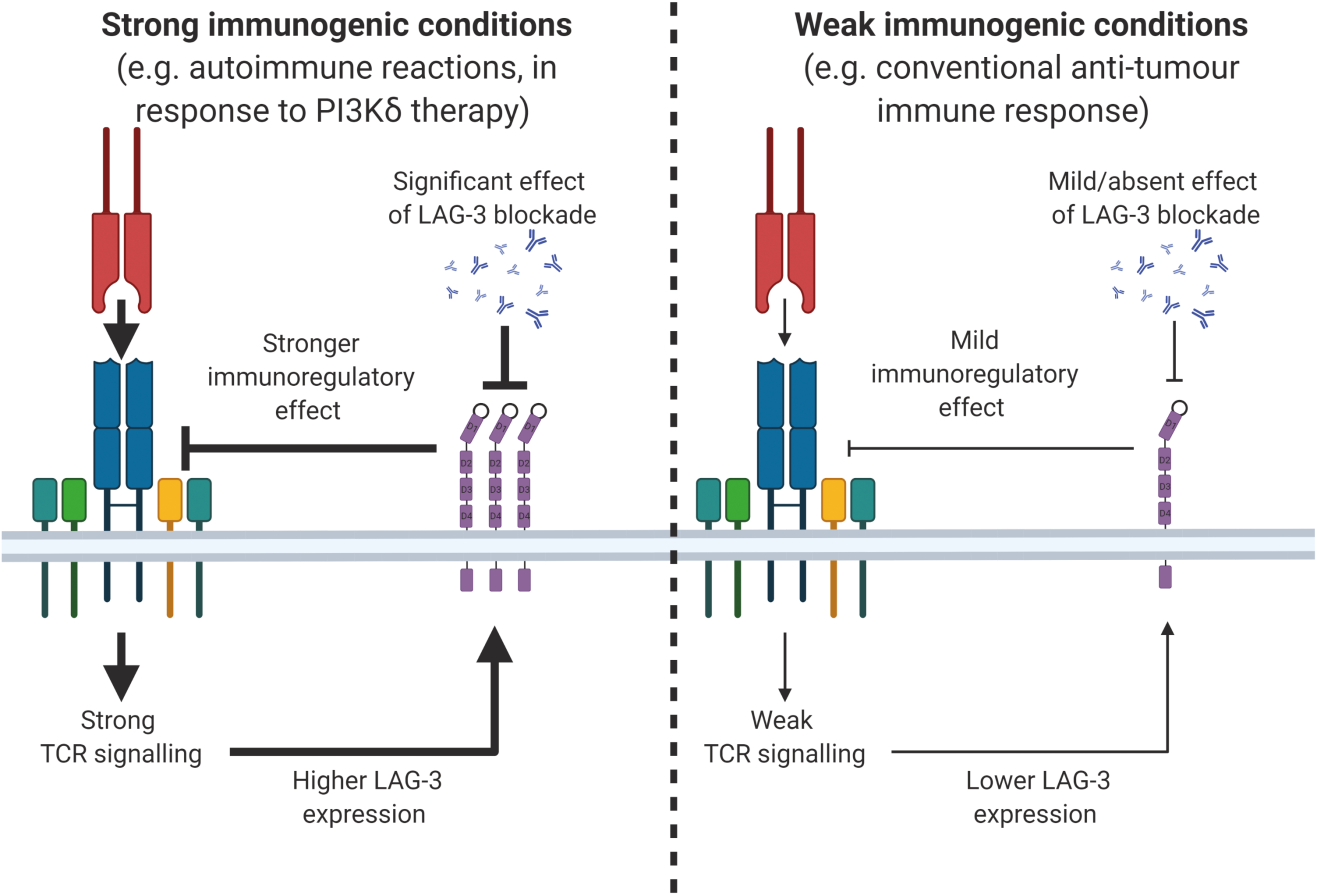
LAG-3 as a rheostat of T-cell immune responses. Immunogenicity may be an important factor that should be considered when assessing the role of LAG-3 in regulating immune responses. Under low immunogenic conditions, LAG-3 expression is likely low and in turn, mild/no effects are observed upon LAG-3 blockade. Instead, during stronger immunogenic conditions during which the role of LAG-3 in immune regulation may be greater, it is possible that a more significant effect is observed upon blockade of LAG-3.

Further clues may also hide within recently made observations that LAG-3^−/−^ mice express higher levels of the co-inhibitory receptors TIM-3, 2B4, and particularly PD-1 on T cells [97]. Similarly, in reverse, knockout of PD-1 results in an upregulation of LAG-3 on T cells, with this effect being more significant for CD8^+^ T cells [97]. These observations may point towards an underlying mechanism for the synergy observed between LAG-3 and PD-1 combinatorial blockade [90]. A recently proposed role of LAG-3 is that it functions as a rheostat of T-cell activation (Fig. 4). This is supported by the observation that the magnitude of LAG-3 expression is proportional to the affinity of a TCR for its pMHC-II complex [98–101]. In keeping with the rheostat role, the inhibitory potential of LAG-3 is proportional to the amount of LAG-3 on the cell surface [38]. It is noteworthy that LAG-3^−^ mice injected with tumour cell lines exhibit minimal survival advantage when compared to WT mice [27, 90, 102]. Similarly, we have recently reported that LAG-3 blockade alone induced no survival advantage in mice with established 4T1 or MC38 tumours [91].

Nonetheless, when directly comparing LAG-3^−/−^ T cells to WT T cells in adoptive transfer experiments, LAG-3^−/−^ T cells display enhanced effector function compared to WT T cells during viral infections [10, 103, 104]. Although the phenotype of LAG-3^−/−^ mice has been underwhelming, non-obese diabetic (NOD)-LAG-3^−/−^ mice develop T-cell-driven autoimmune diabetes very rapidly, and LAG-3^−/−^ mice are more prone to mercury-induced autoimmunity [105–107]. The subtle phenotype observed in LAG-3^−/−^ mice is presumably the result of enhanced effector T-cell function, reduced Treg function, or a combination of both. Immunogenicity might be an important factor to consider in this context. This is exemplified by recent observations made in our lab, where LAG-3 blockade alone did not provide a survival advantage to mice bearing 4T1/MC38 tumours, likely due to low tumour immunogenicity resulting in the absence of an immune response requiring regulation. However, when mice were treated with a small-molecule inhibitor of the PI3Kδ subunit which reduced Treg function and allowed an immune response to develop in some mice, LAG-3 blockade displayed a strongly potentiating effect on anti-tumour immunity [91]. Perhaps, under weak immunogenic conditions (e.g. in the presence of a B16 melanoma [108]) there is no clear LAG-3^−/−^ phenotype. However, under conditions where an immune response requires regulation (e.g. under pro-autoimmune conditions) a LAG-3^−/−^ phenotype emerges.

## Is LAG-3 expressed on epithelium?

Emerging studies have suggested that beyond expressing ligands for co-inhibitory receptors, tumour cells can also express co-inhibitory receptors such as CTLA-4 and PD-1 [109–112]. Interestingly, unlike on T cells, PD-1 signalling on melanoma cells was found to have pro-tumorigenic effects. These findings may reveal unforeseen benefits of checkpoint blockade immunotherapies.

LAG-3 expression has been described on lung cancer cell lines and fresh surgical lung cancer specimens using immunohistochemistry [113]. The results from this study are yet to be corroborated by other laboratories. Two separate studies, one assessing LAG-3 expression on 55 NSCLC cell lines and the other addressing glioblastoma, did not find LAG-3 expression on tumour cells [114, 115]. To add to the opacity surrounding LAG-3 expression on tumour cells, a recent abstract (American Society of Clinical Oncology Meeting 2020) reported expression of LAG-3 on tumour cells from B cell lymphomas as well as a range of epithelial tumours including lung, breast, and ovarian cancers [116]. Furthermore, assessment of RNAseq databases of cancer cell lines like the ‘Expression Atlas’ [117] suggests that various cancer cell lines express at least medium levels (11–1000 transcripts per million) of LAG-3 RNA transcripts, including ­leukaemia (e.g. TALL-1), lymphoma (e.g. A3-KAW), neuroblastoma (e.g. KP-N-YN) glioma (KALS-1), multiple myeloma (e.g. KMS-20), small cell lung carcinoma (e.g. NCI-H2171) and pancreatic carcinoma cell lines (e.g. KP4). It is important, however, to replicate these observations at the protein level before conclusions can be drawn.

Nonetheless, with these conflicting reports in mind, it is unclear what role, if any, LAG-3 plays if expressed on tumour cells. A possible scenario is one in which LAG-3 expression results in pro-tumorigenic effects in a similar fashion to PD-1 [111]. To learn more, further studies are needed which examine LAG-3 expression *in situ* on resected tumours and its association with prognosis.

## Is LAG-3 expressed in the brain?

LAG-3 is potentially expressed in the central nervous system (CNS), initially shown to be expressed in neuronal culture cells [55]. Its exact location remains to be clearly defined and may include neurons and supportive cells such as microglia [118]. Evidence also suggests that LAG-3 is expressed in gliomas with an active inflammatory microenvironment [115, 119, 120]; the modulation of which is being investigated in a current clinical trial (ClinicalTrials.gov Identifier: NCT02658981).

It is emerging that proteins with fundamentally important roles within the immune system are expressed in the CNS where their roles are distinct. For instance, there is evidence that MHC-I molecules help CNS development and plasticity [121]. LAG-3 binding to α-syn has been implicated in Parkinson’s disease (PD) pathogenesis. The cell to cell spread of misfolded α-syn appears to contribute to a group of diseases, including PD, known as α-synucleinopathies [55]. Misfolded pre-formed fibrils (PFFs) of α-syn (rather than monomers) are suggested to be a ligand for LAG-3 where binding leads to endocytosis of the complex, possibly facilitating their cell to cell spread [55, 122–125]. Using the recombinant PFFs [55] and human A53T α-syn models [126], both of which drive α-synucleinopathies in mice, treatment with anti-LAG-3 antibodies or LAG-3^−/−^ knockout resulted in reduced pathology [55, 126]. Further to this, in a recent study investigating the mechanism of pathological α-syn spread, the alkaline surface of the D1 domain of LAG-3 was shown to bind to the acidic C-terminus of α-syn [127]. Serum sLAG-3 levels in PD patients were significantly higher compared to healthy controls [128, 129], indicating that this interaction between LAG-3 and α-syn could provide a possible target for the development of therapeutics designed to slow the progression of α-synucleinopathies.

In another study investigating regional atrophy in PD, LAG-3, and the RAS-related protein, Rab5A were identified as two predictive candidate genes [130]. Interestingly, the previously discussed study identified that LAG-3 facilitated endocytosis of α-syn PFFs and subsequent co-localisation with the early endosomal marker Rab5A [55]. This group hypothesised that LAG-3 and Rab5A control regional propagation of α-syn following initiation of disease in certain brain regions and that α-synucleinopathies spread in stereotypical patterns from one brain region to another [130]. These two studies linking LAG-3 and Rab5A with α-syn aggregation and transfer could be an important starting point in determining whether these proteins are involved in PD initiation and progression.

In contrast, a recent publication by Emmenegger *et al.* found no evidence of LAG-3 in neuronal cell lines, neuronal stem cell-derived cultures nor human brain samples; assaying for expression using multiple anti-LAG-3 antibodies [131]. Marginal LAG-3 expression above background was, however, observed in mouse microglia at the RNA level. It was suggested that the association between LAG-3 and PFFs was due to the promiscuous binding of the PFFs rather than a specific interaction between LAG-3 and α-syn, given α-syn also bound other proteins such as CD4 and tau. It should be noted that Emmenegger *et al.* did not observe the increased survival and delayed α-syn PFF formation in LAG-3 knockout mice reported by *Mao et al.* [55]. Thus, LAG-3 expression in the brain and the implications of the LAG-3 α-syn axis in PD is contentious and the subject of ongoing discussion; further work is required to resolve these conflicting data.

## Conclusion

It is clear that several uncharted territories exist on the map of LAG-3. These will require further exploration if we are to fully exploit LAG-3 as a therapeutic target. We summarise these by raising the following questions:

1. Do different LAG-3 ligands perform distinct roles, and if so, which LAG-3-ligand interactions should be targeted in each scenario?
2. Will developing techniques finally enable the acquisition of the LAG-3 structure? If obtained, what light would this shed on LAG-3 interactions with its ligands or on targeting strategies? Study of LAG-3 via cryo-EM may be achievable as the size barrier is lowered, however, engineering of novel LAG-3 constructs would be beneficial to both cryo-EM and X-ray crystallography approaches.
3. Why does LAG-3 utilise multiple signalling motifs that are not observed in other immune inhibitory receptors? The complexity and uniqueness of LAG-3 signalling leave this fundamental aspect of LAG-3 poorly understood and consequently more difficult to target. Improving our understanding of LAG-3 signalling may provide further downstream targets for therapeutic modulation.
4. Beyond arising as a result of the regulation of LAG-3 expression, does sLAG-3 possess a function that is yet to be identified, and if so, what implications might this have for LAG-3 targeting therapies?
5. Might the LAG-3^−/−^ phenotype hold more subtle traits that have not yet been observed and could this phenotype hold the key to understanding the synergy between LAG-3 and PD-1 combinatorial blockade?
6. Can tumours ectopically express LAG-3, and if so, what implications might this have for LAG-3 targeting therapies? Definition of LAG-3 expression and purpose on tumour cells might reveal a novel insight about LAG-3 function.
7. Is LAG-3 expressed in the brain and if so, on what cells is it expressed? Is it there by chance or as a result of evolutionary motives? It is important to determine whether LAG-3 could be a novel target for the treatment of conditions like PD.

Despite these mysteries, clinical trials targeting LAG-3 remain ongoing. Antibodies aimed at blocking the LAG-3/MHC-II interaction are being tested in five active cancer immunotherapy clinical trials (NCT03365791, NCT03499899, NCT02460224, NCT02061761, NCT02658981) and 15 more are currently recruiting at the time of publication [19]. In addition, an anti-PD-1/LAG-3 bispecific antibody trial is also recruiting (NCT04140500) based on data that antibody targeting of PD-1 and LAG-3 synergistically inhibited tumour growth *in vivo* [90]. So much remains to be answered in the exciting field of LAG-3 biology, and perhaps these empirical experiments framed in clinical trials will offer further key insights: time will tell.

## Data Availability

There are no new data associated with this article.

## Abbreviations

α-syn: α-synucelin
µM: Micro molar
a.a: Amino acid
ADAM10/17: A dis-integrin and metalloproteinase domain-containing protein 10/17
APC: Antigen presenting cells
ATPase: adenosine triphosphatase
BLI: BioLayer interferometry
Ca^2+^: Calcium
CAR-T cell: Chimeric antigen receptor T cell
CEACAM: Carcinoembryonic antigen-related cell adhesion molecule 1
CLRs: C-type lectin receptors
CML: Chronic myelogenous leukemia
CNS: Central nervous system
Cryo-EM: Cryo electron microscopy
CTLA-4: Cytotoxic T-lymphocyte associated protein 4
D1/2/3/4: Domain 1/2/3/4
DCs: Dendritic cells
DC-SIGN: Dendritic cell-specific intercellular adhesion molecule-3-grabbing non-integrin
DSS: Dextran sulphate sodium
Fab: Fragment antigen binding
Fc: Fragment crystallisable
FD: Fibrinogen-like domain
FGL1: Fibrinogen-like protein 1
Gal-3: Galectin 3
GC: Gastric cancer
GFP: Green fluorescent protein
GlcNAc: N-acetyl glucosamine
GnTI: N-acetylglucosaminyltransferase I
GSK3: Glycogen synthase kinase-3
HLA: Human leukocyte antigen
hLAG-3: Human LAG-3
HMGB1: High mobility group protein B1
HNSCC: Head and neck squamous cell carcinoma
IFNγ: Interferon gamma
Ig: Immunoglobulin
IL-2,-12: Interleukin-2, -12
KD: Affinity constant
kDa: Kilo dalton
LAG-3: Lymphocyte activation gene 3
LSECtin: Liver and lymph node sinusoidal endothelial cell C-type lectin
mAb: monoclonal antibody
MHC-II: Major histocompatibility complex class II
mLAG-3: Mouse LAG-3
mRNA: Messenger ribonucleic acid
MS: Multiple sclerosis
Na^+^/K^+^: Sodium/potassium
NK: Natural killer
NOD: Non-obese diabetic
NSCLC: Non-small cell lung cancer
OPCs: Oligodendrocyte precursor cells
PD: Parkinson’s disease
PD-1: Programmed cell death protein 1
PD-L1: Programmed cell death protein ligand 1
pDC: Plasmacytoid dendritic cell
PFF: Pre-formed fibrils
PI3K: Phosphoinositide 3-kinase
pMHC-II: MHC-II protein
PPI: Protein–protein interactions
PtdSer: Phosphatidylserine
Rab5A: RAS-related protein 5A
sLAG-3: Soluble LAG-3
SPR: Surface plasmon resonance
TB: Tuberculosis
TCGA: The cancer genome atlas
TCR: T cell receptor
TIM-3: T cell immunoglobulin and mucin-domain containing-3
TNFα: Tumour necrosis factor alpha
TNM: Tumour, nodes, metastasis
Treg: T regulatory cells
WT: Wild type
